# Familial Robertsonian Translocation Involving Two Autosomes in Swiss Entlebucher Mountain Dogs

**DOI:** 10.3390/ani16203274

**Published:** 2026-10-17

**Authors:** Aldona Pieńkowska-Schelling, Claude Schelling

**Affiliations:** 1 Institute of Genetics, Vetsuisse-Faculty of Bern, University of Bern, 3012 Bern, Switzerland; aldona.pienkowska@icloud.com; 2 Research Platform AgroVet-Strickhof, Vetsuisse-Faculty Zurich, University of Zurich, 8315 Lindau, Switzerland

**Keywords:** Robertsonian translocation, dog, fertility, malformation, chromosome

## Abstract

In domestic animals, chromosomal aberrations are well-documented causes of disorders that result in impaired fertility, malformations, and economic losses. The chromosomes of an alleged sub-fertile bitch of the Entlebucher mountain dog breed should be checked for such aberrations. Metaphase chromosomes were prepared from short-term lymphocyte cultures and microscopically analyzed for numeric or gross structural abnormalities using chromosome banding and chromosome painting probes. The analyses showed that the bitch was a carrier for a Robertsonian translocation involving chromosome 1 and a very small autosome, that could not be identified. The same aberration was detected in three related dogs including a rare case of homozygosity. Human carriers of Robertsonian translocations usually appear normal but seem to have a risk for offspring with genetic imbalances. Much less is known about the consequences of Robertsonian translocations in dogs. Nevertheless, it is of the utmost importance to identify dogs with chromosomal abnormalities and exclude them from breeding in order to prevent the transmission to the next generations. Therefore, the chromosomes of dogs with reproduction problems should always be checked for abnormalities in order to strengthen breeding programs and improve animal welfare.

## Simple Summary

An otherwise healthy bitch of the Entlebucher mountain dog breed caught the attention of her owner because she gave birth to three very small-sized litters. It was suspected that an abnormality in the chromosomes inside her cells might be responsible for this obvious reduction in fertility. The chromosomes of the bitch were evaluated microscopically, and the presence of a specific structural abnormality became obvious, a so-called Robertsonian translocation. In these cases, two chromosomes fuse and form a single chromosome. Dogs that carry this abnormality usually appear normal, but they may produce genetically abnormal sperms or oocytes. A modest reduction in litter size and increased perinatal loss, as well as two harelip cases, were noticed in this pedigree. However, it remains unclear if there is an association between these observations and the Robertsonian translocation. This study revealed that the Robertsonian translocation was inherited over a few generations in a group of Swiss Entlebucher mountain dogs. It is of the utmost importance to recognize the dogs that carry this translocation and exclude them from breeding in order to prevent the transmission to the next generations. Therefore, the chromosomes of suspected dogs should be checked for abnormalities in order to improve animal welfare.

## 1. Introduction

Strong reproductive performance is an important element of any animal breeding program [1]. It ensures high selection potential and genetic progress within populations. In addition to numerous environmental factors, in domestic animals, numerical and structural chromosomal aberrations are well-documented causes of disorders of sexual development (DSDs), impaired fertility, malformations, and economic losses [2,3]. A frequent form of structural chromosomal aberrations in humans and domestic animals is non-homologous Robertsonian translocation (RT) [4,5]. An RT involves the centric fusion of two acrocentric chromosomes, resulting in a bi-armed single chromosome; hence, in a heterozygous animal, the total number of chromosomes is one less than the normal diploid chromosome number [6,7]. If there is no gain or loss of active genetic material, the translocation is called “balanced”, and human and animal carriers of this type of translocation usually appear phenotypically normal [8]. However, they are at an increased risk for fertility problems and offspring with chromosomal imbalances and in the case of humans for uniparental disomy [6,9]. Additionally, Schoemaker et al. demonstrated in a recent human cancer-incidence study that some types of RTs may predispose to elevated risks for certain forms of cancer [10]. RTs are a challenge for meiosis because three chromosomes must synapse and form a trivalent [11]. As a result, the process of meiotic pairing and chromosome segregation during gametogenesis is disturbed, leading to gametes that are genetically unbalanced. Normal gametes are produced if the translocated chromosome disjoins from the homologous chromosomes. All other types of disjunctions lead to unbalanced gametes and aneuploid embryos that are not viable or show serious malformations. Review articles provide detailed information on the molecular mechanisms involved in the formation of RTs [6,7]. All autosomes of the domestic dog [12] and domestic cattle [13] are acrocentric and prone to RTs. However, in dogs, only a subset of autosomes has been shown to be involved in RTs. This is in contrast to cattle, for which spontaneous cases of RTs have been reported for all 29 autosomes. An important task of any breeding program is the identification and elimination of animals that carry chromosomal aberrations. It is not clear whether chromosomal aberrations in domestic dogs are common and to what extent they are clinically important. Notably, among breeders and veterinarians, the awareness of cytogenetic analyses in companion animals, which enable both optimized diagnosis and prognosis, is poor [14]. Several factors are responsible for this. The number of laboratories capable of performing such analyses is declining, and breeders’ or owners’ economic concerns prevent cytogenetic testing [3,15]. Carriers of chromosome abnormalities are often found incidentally when animals are surgically sterilized. Additionally, fertility problems, behavior problems, or ambiguous external genitalia may prompt an owner to bring their dog to a veterinarian. Whatever the reasons, publications reporting chromosomal aberrations in dogs are rare, compared to the literature on humans. Animal welfare and protection laws forced a change in dog breeding from beauty and exhibitions to health and genetic diversity. Dog breeding clubs are often overwhelmed by new approaches to genetics (gene tests, genetic diversity measuring, etc.). An otherwise healthy female Entlebucher mountain dog (EMD) was suspected to carry a chromosomal abnormality, which may have been responsible for her subfertility. The main goal of this study was to evaluate the chromosomes of this animal and three related dogs.

## 2. Materials and Methods

### 2.1. Animals

**Figure 1:**
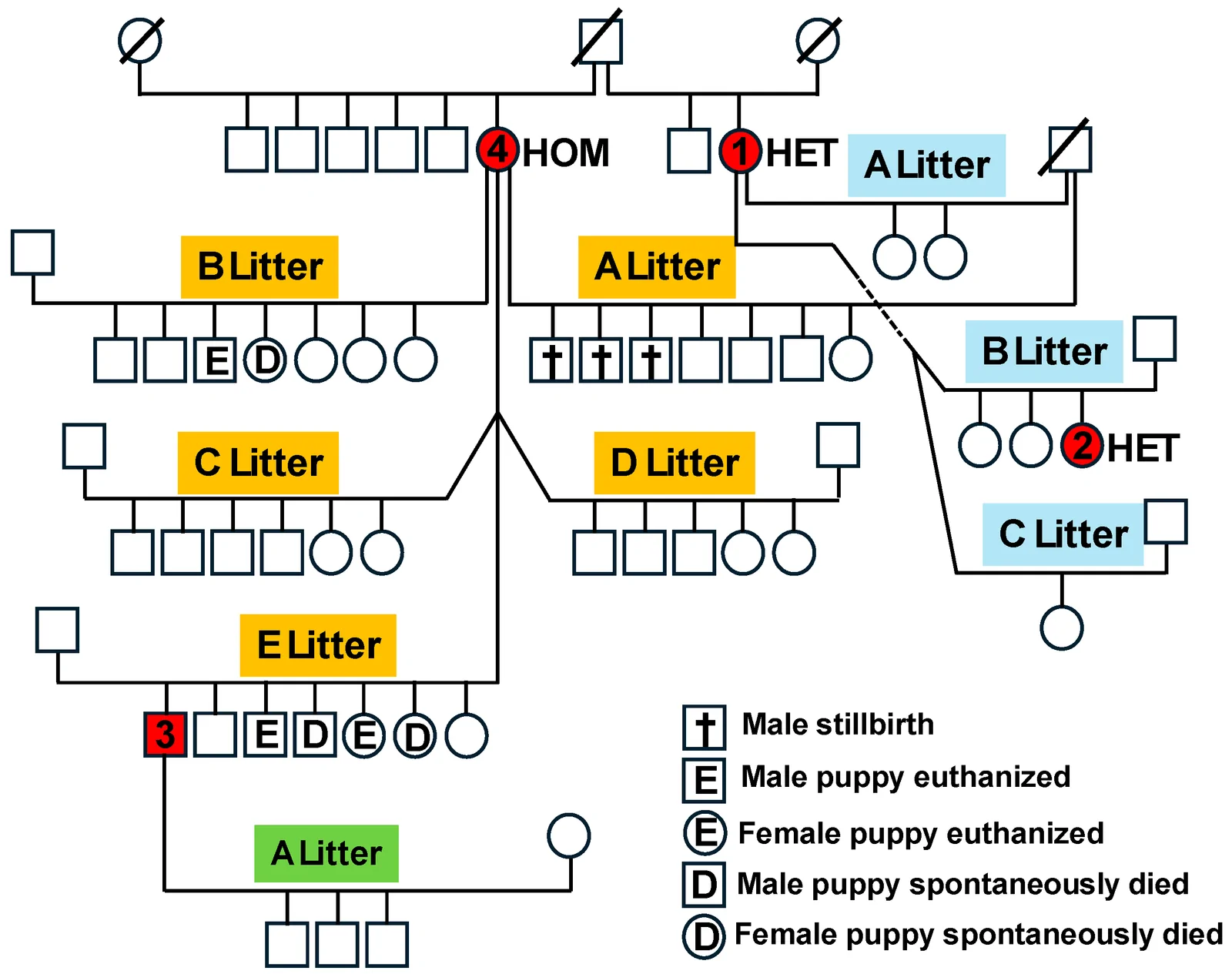
This figure shows a partial pedigree of Entlebucher mountain dogs showing familial transmission of the Robertsonian translocation. Squares and circles indicate male and female dogs, respectively. Symbols with a slash represent dead animals. Bitch 1, bitch 2, and sire 3 were found to be heterozygous for the Robertsonian translocation. Bitch 4, the mother of sire 3, was found to be homozygous for the Robertsonian translocation. The ancestors of bitch 1 and bitch 4 were already dead, and their chromosomes could not be analyzed. Because of missing consent and/or economic concerns, the other six sires and one bitch in the pedigree could not be analyzed. Bitch 1 had three very small litters, while bitch 4 had five well-sized litters. However, she had an extremely high rate of puppy losses (28%), including stillborn puppies (†), puppies that had to be euthanized (E) and puppies that died spontaneously (D). One male puppy each in litters B and E was euthanized because of congenital harelip. The reason for euthanization of a female puppy in litter E was not clear, and no detailed information on the stillborn puppies and those that died spontaneously was available.

One female EMD (bitch 1) caught the attention of her owner as she had given birth to three litters with one, two, and three puppies, respectively. Because the average litter size in this population was 5.8 puppies, it was suspected that the otherwise healthy bitch might be sub-fertile, and the owner requested a cytogenetic evaluation. Bitch 2, a daughter of bitch 1, was a breeding candidate, and, in addition to the breeding soundness exam, her owner requested that the chromosomes be evaluated as well. Sire 3 was an active breeding animal and investigated because of his relationship to bitch 1. Because he carried a similar aberration, his mother (bitch 4) was analyzed as well. Relationships between the four dogs were determined via our EMD-databank and are provided in Figure 1.

### 2.2. Cytogenetic Analyses

The dogs’ heparinized blood samples were collected in private veterinary practices and sent to our laboratory. Following short-term lymphocyte culturing, metaphase chromosomes were prepared following the modified protocol of Arakaki and Sparkes [16]. The modifications involved a longer colcemide treatment (2 h) and precooling the freshly mixed fixative solution (glacial acetic acid methanol 1:3) at −30 °C. After dropping aliquots onto the slides, chromosomes were stained with 4′,6-diamidino-2-phenylindole (DAPI) according to the manufacturer’s protocol (ThermoFisher, Reinach, Switzerland). DAPI-staining is comparable to GTG-banding but less time consuming. An RT painting probe of the translocated chromosome was developed following an in-house protocol. Briefly, several copies of the translocated chromosome were micro-dissected under an inverted microscope using Duran^®^ glass needles attached to a micromanipulator (Zeiss, Hombrechtikon, Switzerland). After two rounds of degenerate oligo-primed polymerase chain reaction (PCR) using a hand-mixed Telenius primer (5’CCGACTCGAGNNNNNNATGTGG3’) [17], amplified DNA was labeled with Fluorescein-12-dUTP or Aminoallyl-dUTP-Texas-Red (Jena Bioscience, Jena, Germany) by degenerate oligo-primed PCR and applied for fluorescence in situ hybridization (FISH) experiments [18]. For each dog, more than 100 metaphases were analysed to ensure a 97% chance of detecting a second cell line [19]. Pictures were captured by a fluorescence microscope (Zeiss Axioimager Z1, Zeiss, Hombrechtikon, Switzerland) using a CoolCube CCD camera (Princeton Instruments, Princeton, NJ, USA) and were evaluated using IKAROS V 5.4 software (MetaSystems, Altlußheim, Germany).

## 3. Results

**Figure 2:**
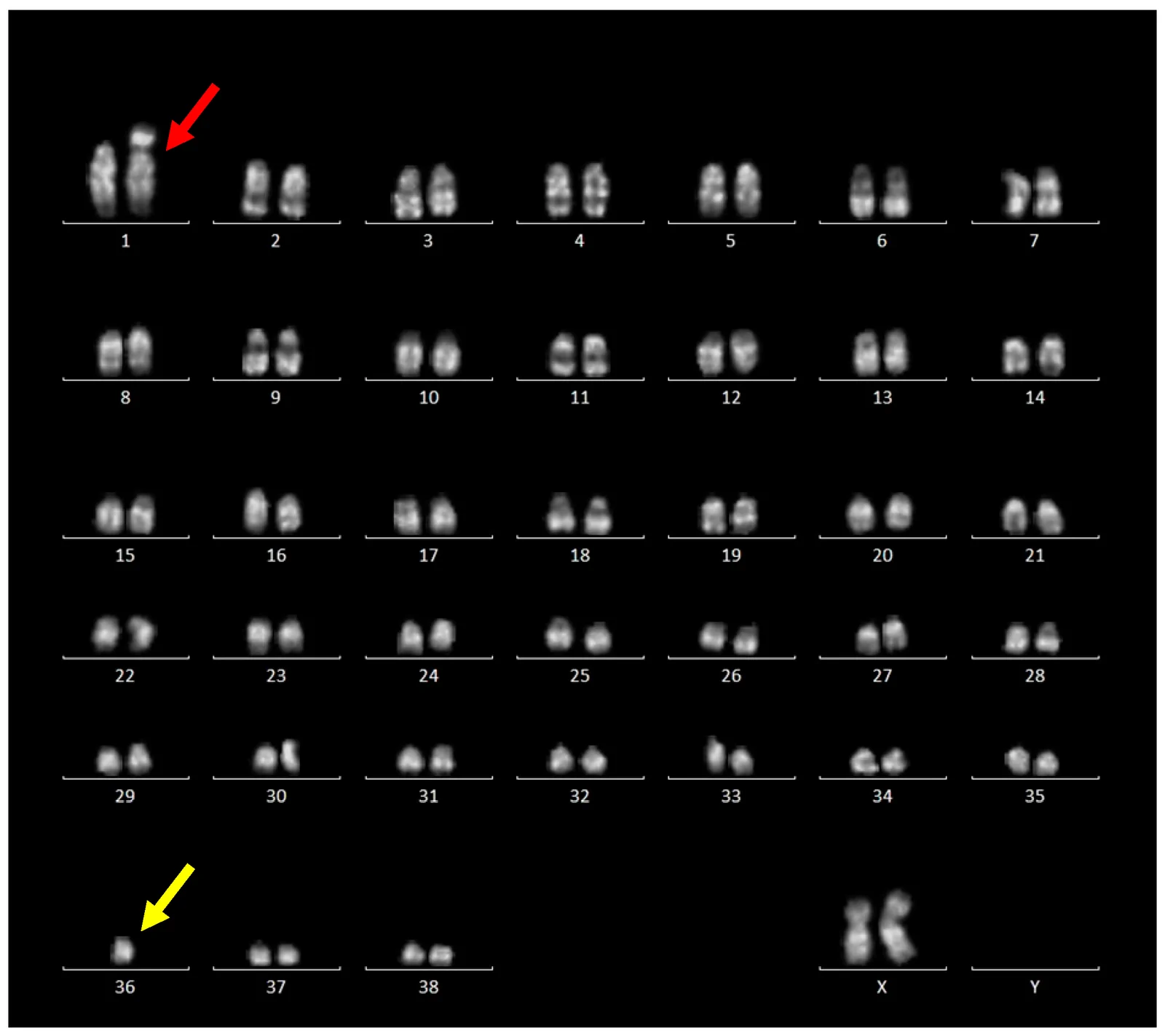
This figure shows the chromosomes of bitch 1 after treatment using the blue DNA fluorescent stain 4′,6-diamidino-2-phenylindole (DAPI), arranged in homologous pairs from largest to smallest, with the sex chromosomes placed last. Red arrow points at the translocated chromosome consisting of *Canis familiaris* autosome 1 and a very small not identified autosome, tentatively chromosome 36. Yellow arrow points at a possible homolog of the unidentified autosome.

**Figure 3:**
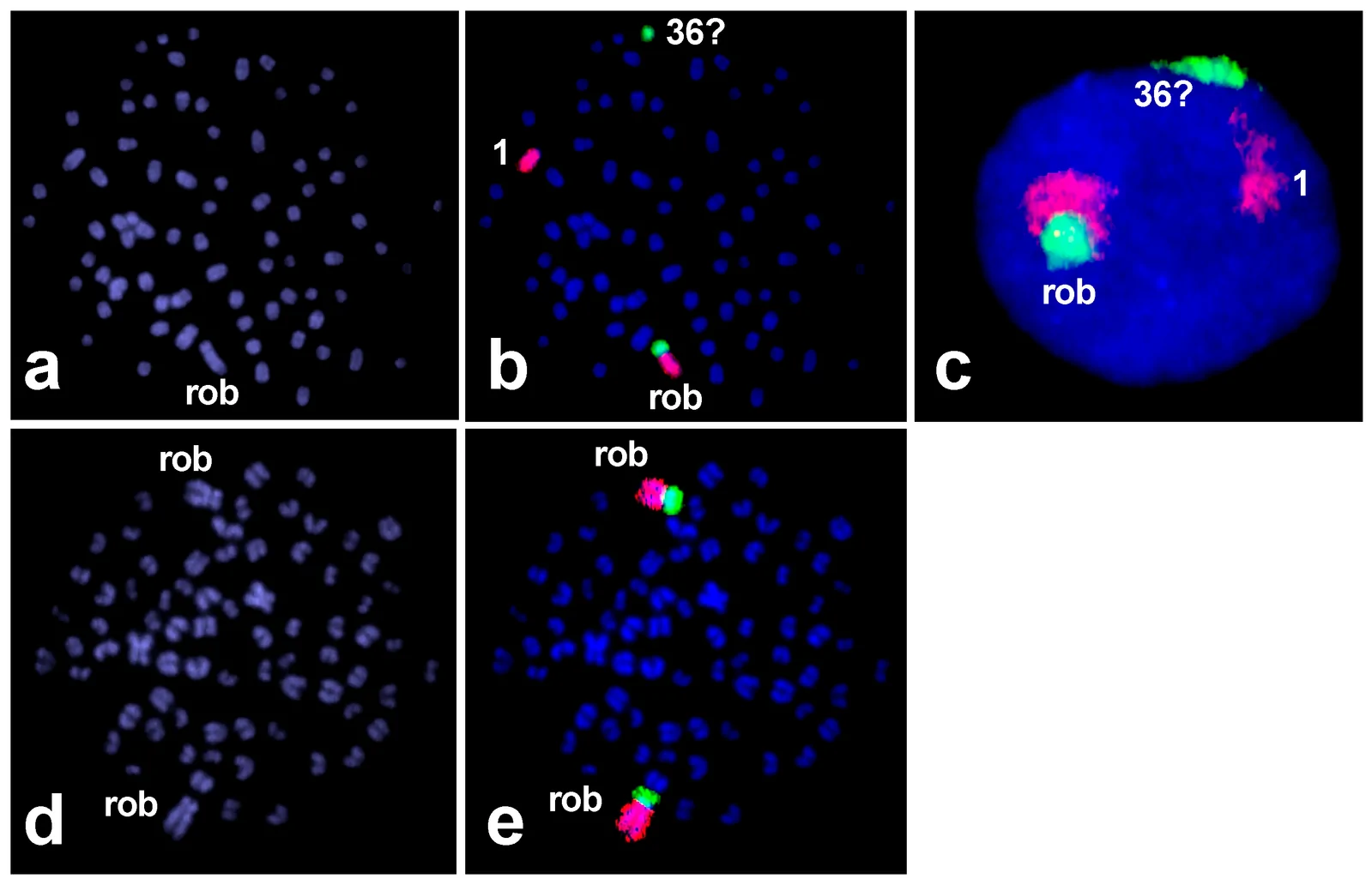
(**a**,**b**) show the same metaphase of bitch 1 (heterozygous for RT) before (4′,6-diamidino-2-phenylindole (DAPI) and after fluorescence in situ hybridization with the Robertsonian translocation painting probe, respectively. Comparatively, (**d**,**e**) show the results for bitch 4 (homozygous for Robertsonian translocation). (**c**) shows clear hybridization signals of the Robertsonian translocation painting probe in an interphase nucleus of bitch 1. Green color and red color are the epiluminescence of fluorescein-12-dUTP (channel green) and aminoallyl-dUTP-Texas-Red (channel red), respectively.

After staining the chromosomes of bitch 1 (proposita) with Giemsa, it became clear that there were only 77 chromosomes present in all cells analyzed. In contrast to the normal female canine karyotype (78,XX) with 76 acrocentric autosomes and two sub-metacentric X chromosomes, bitch 1 had two X chromosomes, one bi-armed derivative and 74 free autosomes. It was suspected that an RT had occurred and led to a chromosome complement of 77 chromosomes. Based on analysis of numerous DAPI-stained metaphases and a few karyograms, it was suggested that a very large and a very small autosome were involved in this non-homologous mono-centric RT (Figure 2 and Figure 3). Initially, it was considered that this heterozygous carrier was an isolated case and that banning her from breeding would be the end of the story. But then bitch 2, a daughter of bitch 1, passed the exam for breeding soundness and became a breeding candidate. Before she was used for breeding, the owner wanted her chromosomes to be checked.

We then developed a specific chromosome painting probe for this translocation (RT-painting probe), allowing for easy diagnostics using FISH technology. It could be shown that the RT was transmitted from bitch 1 to bitch 2 and present in all cells. Sire 3, related to bitch 1 had sired one small-sized litter (litter size at birth: three puppies) and was found to be heterozygous for the same RT Like the other carriers he was banned from breeding. His mother (bitch 4) turned out to be homozygous for the RT (Figure 3d,e) and had given birth to five normal-sized litters (Figure 1).

All four animals carrying the RT appeared healthy and phenotypically normal. Bitch 1 had three litters (A–C) with very small litter sizes at birth but no puppy losses were registered. Bitch 4 was homozygous for the RT and had five litters with a total of 32 puppies. The average litter size at birth was 6 puppies, slightly above the actual population average for litter size at birth with 5.8 puppies. However, the rate of puppy losses (six males and three females) amounted to 28%. For litters C and D no puppy losses were recorded by the breeder. For litters A, B and E a total of three stillborn, three euthanized and three puppies that died spontaneously were recorded. Unfortunately, the records of the breeder showed only the sex but did not reveal the cause of the puppy losses. Only for two male puppies, harelip was listed as the cause for euthanasia. Sire 3 sired only one litter with three puppies, was found to be a carrier too and excluded from breeding.

## 4. Discussion

Chromosomal disorders are not part of the daily routine in a veterinary practice. Consequently, few clinical cases make it to the laboratory. Although the reduced number of chromosomes in canine RTs makes their detection easy, only a few cases have been published because the carriers are not recognized. We identified a probably balanced non-homologous RT in a group of four related EMDs. Because of financial and club-internal political concerns, it was not possible to analyze more EMDs and exactly determine the origin and frequency of this RT. Nevertheless, a familial transmission of the RT seems plausible. The presence of this structural aberration may explain the observed reduction in fertility of the female proposita. Being a heterozygous carrier of an RT predisposes, among other things, to the production of unbalanced gametes and aneuploid offspring; hence, the reduced fertility (litter size) of the affected animals. If a breeding dog is suspected to be sub- or infertile, the animal is usually banned from breeding without contacting a veterinarian, and the chromosomes are not checked. Experienced dog breeders recognize “problem bitches” rather early and precisely. After screening Golden and Labrador retriever records for the period of nine years, we identified only five female candidates that had at least three litters with three or fewer puppies. Four of them had already been recognized by the breeder and banned from breeding (not published). In the present case, the owner of an otherwise healthy bitch suspected subfertility because she had given birth to three litters (three different sires) with a litter size at birth clearly below average. After consulting with us and recognizing possible consequences for his kennel, he ordered a cytogenetic analysis.

The majority of published canine RTs described isolated cases, and only three publications reported a familial transmission [20,21,22]. The RT presented here involved CFA1 and one of the smallest autosomes. For a long time, the identification of canine autosomes involved in RT was a challenge because of the complex karyotype. In addition to the high number of chromosomes, all autosomes are acrocentric and gradually get shorter from CFA1 to 38. Only autosomes CFA1 to 21 may be identified by traditional banding techniques [12]. CFA22 to 38 should be identified using FISH methodology via BAC clones or chromosome painting probes [23]. As a result, when this methodology was not yet available, publications on RT often did not include autosome identification. Because of internal difficulties, we could not perform any FISH experiments using BAC clones and had to rely on classical cytogenetic analyses for the characterization of the present RT. The large autosome involved in the RT is most likely CFA1 because of its size. CFA1 has been part of at least two canine RT cases published in earlier studies [24,25]. Based on the DAPI-staining alone, we could not identify the smaller autosome.

RT homozygosity is extremely rare in humans [26] and in dogs [22,25]. In 2010, just ten human cases were identified in the literature. Almost all cases arose from inbreeding in families without any associated pathology but with impaired fertility. Since then, more cases have been published (e.g., [27,28]). However, it remains unclear whether RT homozygosity in humans is in fact so exceptional or if it is just under-represented due to missing phenotypic irregularities. The parents of bitch 4 are related (EMD-databank), and both may have passed down the translocated chromosome to bitch 4; this might explain her homozygous status. Alternatively, bitch 4 might have inherited both copies of the translocated chromosome from her father, representing a case of uniparental disomy. However, this mechanism has not yet been reported for dogs.

Perinatal mortality rates in dogs are high and require greater attention from breeders and veterinarians [29]. Bitch 4, homozygous for RT, gave birth to 32 puppies with puppy losses amounting to 28%. However, it remains unclear if this RT is connected to this puppy loss. It was difficult to compare our observations with results from other publications, as there is almost no data available. Considering all published cases of heterozygous and homozygous canine RTs together, information for 51 dogs (45 heterozygous and 6 homozygous) was available [20,21,22,25]. With the exception of two homozygous females (both with inguinal hernias) and one heterozygous female (absent puberty), all dogs appeared to be phenotypically normal. These three reported abnormal phenotypes are relatively common in dogs, and whether a true association with RT exists remains unclear. The same is relevant for the present RT. The causes of these dramatic puppy losses in three litters of bitch 4 remain unknown.

In the near future, we recommend observing litter sizes, females failing to get pregnant, and sires with low reproduction success in order to identify and eliminate all carriers of chromosomal abnormalities.

## 5. Conclusions

Dogs with chromosome abnormalities should be identified early and excluded from breeding programs. Chromosome analysis remains the principal test for disorders of reproduction in domestic animals, even though the awareness among breeders and vets for such analyses is poor. The detection of RT and other structural aberrations using BAC clones or chromosome painting probes is straightforward but costly. Unfortunately, when the breeders received the information about this “genetic” problem, they preferred to ignore it and protect their breeding dogs. This behavior prevented an estimate of the frequency of RT in this population. Despite major efforts, it was not possible to identify the small autosome via molecular genetic methodology. If dogs carrying chromosomal abnormalities are not banned from breeding, they may transmit the aberrations to the next generation and thereby reduce fertility in the population. Human carriers of RT have, among other things, an increased risk for fertility problems and offspring with miscarriages, uniparental disomy, and non-viable offspring. Although the pedigree of the present study is compatible with reduced fertility in some carriers, the presence of miscarriages and uniparental disomy remains speculative. Even if the number of dogs with chromosomal aberrations is presumably small and no direct gross impact is visible, they deserve special attention because they might be plagued by disorders and diseases as side-effects of their chromosomal abnormality. The weight of animal welfare should count more than economic concerns.

## Data Availability

The raw data supporting the conclusions of this article will be made available by the authors on request.

## Abbreviations

The following abbreviations are used in this manuscript:

CFA	*Canis familiaris* autosome
BAC	Bacterial artificial chromosome
DAPI	4′,6-diamidino-2-phenylindole
EMD	Entlebucher mountain dog
FISH	Fluorescence in situ hybridization
PCR	Polymerase chain reaction
RT	Robertsonian translocation

## References

[1] Eldridge F.E. (1985). Cytogenetics of Livestock.

[2] Raudsepp T., Chowdhary B.P. (2016). Chromosome aberrations and fertility disorders in domestic animals. Annu. Rev. Anim. Biosci..

[3] Lewis N.M., Canedo-Ribeiro C., Rathje C.C., Jennings R.L., Danihel M., Bosman L.M., Silvestri G., Griffin D.K. (2022). The economic burden of chromosome translocations and the benefits of enhanced screening for cattle breeding. Animals.

[4] Zhao W.-W., Wu M., Chen F., Jiang S., Su H., Liang J., Deng C., Hu C., Yu S. (2015). Robertsonian Translocations: An Overview of 872 Robertsonian Translocations Identified in a Diagnostic Laboratory in China. PLoS ONE.

[5] Jiménez J.M., Morales R., Molina A., Moreno-Millán M., Demyda-Peyrás S. (2022). Effect of the rob(1;29) translocation on the fertility of beef cattle reared under extensive conditions: A 30-year retrospective study. Reprod. Domest. Anim..

[6] Poot M., Hochstenbach R. (2021). Prevalence and phenotypic impact of Robertsonian translocations. Mol. Syndromol..

[7] de Lima L.G., Guarracino A., Koren S., Potapova T., McKinney S., Rhie A., Solar S.J., Seidel C., Fagen B.L., Walenz B.P. (2025). The formation and propagation of human Robertsonian chromosomes. Nature.

[8] Benn P., Merrion K. (2024). Chromosome segregation of human nonhomologous Robertsonian translocations: Insights from preimplantation genetic testing. Eur. J. Hum. Genet..

[9] Liehr T. (2010). Cytogenetic contribution to uniparental disomy (UPD). Mol. Cytogenet..

[10] Schoemaker M.J., Jones M.E., Higgins C.D., Wright A.F., Swerdlow A.J., the UK Clinical Cytogenetics Group (2019). Mortality and cancer incidence in carriers of constitutional t(11;22)(q23;q11) translocations: A prospective study. Int. J. Cancer.

[11] Gardner R.J.M., Sutherland G.R., Shaffer L.G. (2012). Chromosome Abnormalities and Genetic Counseling.

[12] Świtoński M., Reimann N., Bosma A.A., Long S., Bartnitzke S., Pieńkowska A., Moreno-Milan M.M., Fischer P. (1996). Report on the progress of standardization of the G-banded canine (*Canis familiaris*) karyotype. Chromosome Res..

[13] Iannuzzi A., Parma P., Iannuzzi L. (2021). Chromosome abnormalities and fertility in domestic bovids: A review. Animals.

[14] Neuhauser S., Handler J., Schelling C., Pieńkowska-Schelling A. (2018). Disorder of sexual development in a mare with an unusual tentative mosaic karyotype: 63,X/64Xdel(Y). Sex. Dev..

[15] Basrur P.K., Stranzinger G. (2008). Veterinary cytogenetics: Past and perspective. Cytogenet. Genome Res..

[16] Arakaki D.T., Sparkes R.S. (1963). Microtechnique for Culturing Leukocytes from Whole Blood. Cytogenetics.

[17] Telenius H., Carter N.C., Bebb C.E., Nordenskjöld M., Ponder B.A.J., Tunnacliffe A. (1992). Degenerate oligonucleotide-primed PCR: General amplification of target DNA by a single degenerate primer. Genomics.

[18] Pieńkowska-Schelling A., Zawada M., Schelling C. (2005). A canine X chromosome painting probe applied to four canid species: Close relationship of a heterochromatic-like sequence between the dog and the blue fox. J. Anim. Breed. Genet..

[19] Hook E.B. (1977). Exclusion of chromosomal mosaicism: Tables of 90%, 95%, and 99% confidence limits and comments on use. Am. J. Hum. Genet..

[20] Stone D.M., Mickelsen W.D., Jacky P.B., Prieur D.J. (1991). A novel Robertsonian translocation in a family of Walker hounds. Genome.

[21] Larsen R.E., Dias E., Cervenka J. (1978). Centric fusion of autosomal chromosomes in a bitch and offspring. Am. J. Vet. Res..

[22] Larsen R.E., Dias E., Flores G., Selden J.R. (1979). Breeding studies reveal segregation of a canine Robertsonian translocation along mendelian proportions. Cytogenet. Cell Genet..

[23] Breen M. (2008). Canine cytogenetics—From band to basepair. Cytogenet. Genome Res..

[24] Nogueira D.M., Armada J.L.A., Penedo D.M., Tannouz V.G.S., Meyers-Wallen V.N. (2019). Persistent Mullerian Duct Syndrome in a Brazilian Miniature Schnauzer Dog. An. Acad. Bras. Ciênc..

[25] Mayr B., Krutzler J., Schleger W., Auer H. (1986). A new type of Robertsonian translocation in the domestic dog. J. Hered..

[26] O’Neill I.D. (2010). Homozygosity for constitutional chromosomal rearrangements: A systematic review with reference to origin, ascertainment and phenotype. J. Hum. Genet..

[27] Ouertani I., Chaabouni H. (2025). Familial transmission of a Robertsonian translocation rob(21;22): A case report. Glob. J. Med. Clin. Case Rep..

[28] Sahraeean S., Jebelli A., Shahbazi Z., Piryaei F. (2023). Homozygosity for Robertsonian Translocation (14q;15q) in a Newborn with a Familial History of Recurrent Abortion and Newborns Affected by Hepatosplenomegaly: A Case Report. J. Reprod. Infertil..

[29] Veronesi M.C., Fusi J. (2023). Biochemical factors affecting newborn survival in dogs and cats. Theriogenology.

